# A panel of selected serum protein biomarkers for the detection of aggressive prostate cancer

**DOI:** 10.7150/thno.55676

**Published:** 2021-04-15

**Authors:** Jin Song, Shiyong Ma, Lori J. Sokoll, Rodrigo V. Eguez, Naseruddin Höti, Hui Zhang, Phaedre Mohr, Renu Dua, Dattatraya Patil, Kristen Douglas May, Sierra Williams, Rebecca Arnold, Martin G. Sanda, Daniel W. Chan, Zhen Zhang

**Affiliations:** 1 Center for Biomarker Discovery and Translation, Department of Pathology, Johns Hopkins University School of Medicine, Baltimore, MD, 21287, USA.; 2 Department of Oncology, Johns Hopkins University School of Medicine, Baltimore, MD, 21287, USA.; 3 Department of Urology, Johns Hopkins University School of Medicine, Baltimore, MD, 21287, USA.; 4 Department of Radiology, Johns Hopkins University School of Medicine, Baltimore, MD, 21287, USA.; 5 Department of Urology, Emory University School of Medicine, Atlanta, GA, 30322, USA.

**Keywords:** prostate, prostate neoplasms, prostate-specific antigen, biomarker, aggressive

## Abstract

**Background:** Current PSA-based tests used to detect prostate cancer (PCa) lack sufficient specificity, leading to significant overdetection and overtreatment. Our previous studies showed that serum fucosylated PSA (Fuc-PSA) and soluble TEK receptor tyrosine kinase (Tie-2) had the ability to predict aggressive (AG) PCa. Additional biomarkers are needed to address this significant clinical problem.

**Methods:** A comprehensive Pubmed search followed by multiplex immunoassays identified candidate biomarkers associated with AG PCa. Subsequently, multiplex and lectin-based immunoassays were applied to a case-control set of sera from subjects with AG PCa, low risk PCa, and non-PCa (biopsy negative). These candidate biomarkers were further evaluated for their ability as panels to complement the prostate health index (*phi*) in detecting AG PCa.

**Results:** When combined through logistic regression, two panel of biomarkers achieved the best performance: 1) *phi,* Fuc-PSA, SDC1, and GDF-15 for the detection of AG from low risk PCa and 2) *phi*, Fuc-PSA, SDC1, and Tie-2 for the detection of AG from low risk PCa and non-PCa, with noticeable improvements in ROC analysis over *phi* alone (AUCs: 0.942 vs 0.872, and 0.934 vs 0.898, respectively). At a fixed sensitivity of 95%, the panels improved specificity with statistical significance in detecting AG from low risk PCa (76.0% vs 56%, *p*=0.029), and from low risk PCa and non-PCa (78.2% vs 65.5%, *p*=0.010).

**Conclusions:** Multivariate panels of serum biomarkers identified in this study demonstrated clinically meaningful improvement over the performance of *phi*, and warrant further clinical validation, which may contribute to the management of PCa.

## Introduction

Prostate cancer (PCa) is the most common non-cutaneous solid tumor in men and has a high prevalence among men aged 50 years and above in the United States. In 2021, new cases are estimated at 248,530 with approximately 34,130 deaths 1. The serum test for prostate-specific antigen (PSA) was developed and approved by the Food and Drug Administration (FDA) for prostate cancer over 30 years ago 2. While PSA has become a routine clinical test, PSA screening has garnered substantial criticism in recent years due to the potential for overdetection and overtreatment of PCa. In particular, recommendations by the United States Preventive Services Task Force (USPSTF) 3 have generated significant debate regarding PSA-based screening. Biopsies trigged by a marginally elevated serum PSA level or other reason will likely result in a significant number of biopsy-positive cases for whom the majority will have low risk disease that may not require active clinical intervention. Overtreatment could be mitigated with a diagnostic test capable of identifying aggressive (AG) PCa prior to biopsy. While there is no consensus on the definition of “aggressiveness,” it is generally agreed that Gleason score (GS) is likely the best indicator. In general, higher GSs are associated with more aggressive PCa defined in terms of disease-free survival 4, 5. The most widely accepted histological cutoff for PCa is GS 7. When the GS is 7 or higher, the tumor is considered “aggressive”.

The goal of this study was to identify and combine serum proteomic biomarkers into a panel for distinguishing AG PCa from low risk cancer. Two biomarkers, fucosylated PSA (Fuc-PSA) and soluble TEK receptor tyrosine kinase (Tie-2), were discovered in our previous Early Detection Research Network (EDRN) studies with demonstrated ability to predict AG PCa 6-8. Fucosylated proteins have been found to be associated with cancer and potentially used as tumor markers 9-11. An example is the fucosylated alpha-fetoprotein (AFP-L3), an FDA cleared diagnostic test for assessing the risk of developing hepatocellular carcinoma 12, 13. We developed quantitative lectin-based immunoassays for serum Fuc-PSA and demonstrated that Fuc-PSA could be an effective biomarker to detect AG PCa 7, 8. Serum angiogenic factors are potential candidates for prognostic biomarkers in PCa 14, 15. Tie-2 is a transmembrane tyrosine kinase receptor for angiopoietins and is crucial for angiogenesis and vascular maintenance 16, 17. We previously demonstrated that serum levels of Tie-2 were elevated in PCa patients with GS 8-10 6. In this study, we evaluated whether combinations of Fuc-PSA, Tie-2, and/or other selected biomarkers from an expanded list of candidates combined with current FDA approved PSA-based test modalities, specifically, prostate health index (*phi*) 18, could improve their diagnostic ability for the detection of AG PCa.

## Materials and Methods

### Study Design

We performed a comprehensive literature search and identified 22 additional candidate biomarkers reported to be associated with AG PCa. We tested these 22 candidates with multiplex immunoassays in a well-characterized in-house clinical sample set. Based on bootstrap area-under-curve (AUC) analysis, the list was reduced to the 10 best performing biomarkers with respect to the combined criteria of a relatively high AUC mean and a relatively low AUC standard deviation (STD) in separating low risk versus AG PCa. In this study, using a case-control sample set, we evaluated whether these 10 biomarkers as well as Tie-2 and Fuc-PSA in combined use with current FDA approved PSA-based test modalities, specifically, prostate health index (*phi*) 18, could further improve the detection of AG PCa.

### Specimens

Specimens for this study were collected at Beth Israel Deaconess Hospital, Harvard Medical School from 2005 to 2008 as part of the prospective EDRN Clinical Validation Center cohort 19. Eligibility for the EDRN cohort included patient age greater than 40 years, no prior prostate surgery, biopsy or history of PCa, availability of serum samples with corresponding clinical data, and completion of biopsy under transrectal ultrasound guidance using a standard template after enrolment. Serum samples were collected prior to initial biopsy and stored at -80 ºC until analysis. Serum samples obtained from 90 patients, including 60 patients with histologically diagnosed PCa and 30 biopsy negative controls were included in this study with institutional approval. For the current study, GS was used as a surrogate for PCa aggressiveness. Consistent with the majority view in the literature 4, 5, 20, a tumor with a GS 7 or greater was considered as AG PCa and GS 6 or less as low risk PCa.

### Reagents

Human Magnetic Luminex Assays (LXSAHM-15, LXSAHM-08, and LXSAHM-02) were purchased from R&D Systems (Minneapolis, MN). Magnetic COOH beads, amine coupling kits, and Bio-Plex Pro Reagent kits were purchased from Bio-Rad Laboratories (Hercules, CA). NHS and Sulfo-NHS, EDC, EZ-Link^TM^ Sulfo-NHS-Biotin, and Zeba^TM^ Spin Desalting Columns were purchased from Thermo Scientific (Rockford, IL). Agarose bound *Aleuria Aurantia Lectin* (AAL) was purchased from Vector Laboratories (Burlingame, CA). Pierce™ BCA Protein Assay Kit was purchased from Thermo Fisher Scientific (Waltham, MA).

### Multiplex immunoassays

Human Magnetic Luminex Assays were performed following the manufacturer's protocols on the Bio-Plex 200 system. Samples were diluted 1:2 (the initial 15-plex and the finalized 8-plex assays) or 1:50 (2-plex assay) in the calibrator diluent. Calibration curves were established using 7 calibrators in a 3-fold dilution series in the calibrator diluent derived from a mixture of the highest standard points of multiple recombinant proteins. The highest standards were 215.8, 883.2, 4.8, 25.6, 63.4, 1977.9, 169.7, and 10.3 ng/mL for CD276 molecule (B7-H3), phospholipase A2 group VII (PLA2G7), growth differentiation factor 15 (GDF-15), interleukin-6 receptor subunit alpha (IL-6 R alpha), Syndecan-1 (SDC1), vascular cell adhesion molecule 1 (VCAM-1), TEK receptor tyrosine kinase (Tie-2), and interleukin 16 (IL-16), respectively (8-plex); 40 U/mL and 57.2 ng/mL for cancer antigen (CA 15-3) and matrix metallopeptidase (MMP-2), respectively (2-plex). Heat shock 27 kDa protein (HSP27) assay (1-plex) was carried out with the sample diluted 1:4 in the standard diluent, and the calibration curve was established using 7 calibrators in 2.5-fold dilution series in the standard diluent. The highest standard of the recombinant protein in the assay was 3.0 ng/mL. Immunoassays were performed in duplicate on 96-well Bio-Plex flat bottom plates. All samples were randomized with respect to their plate locations.

Calibration curves were constructed with Bio-Plex Manager Software version 6.1.1 using a 5-parametric (5-PL) nonlinear logistic regression curve fitting model. Assay sensitivity (limit of blank, LOB) was defined as the concentration of analyte corresponding to the median fluorescent intensity (MFI) of the background plus two STDs of the mean background MFI. Intra-assay precision was calculated as the coefficient of variance (%CV) on 4 replicates of pooled normal sera (S7023 from Sigma-Aldrich) on a single assay plate. Inter-assay precision was calculated as the %CV from 3 replicates. The assay working dynamic range was defined as the range between the lower limit of quantification (LLOQ) and the upper limit of quantification (ULOQ) in which an assay is both precise (intra-assay %CV ≤10% and inter-assay %CV ≤15%) and accurate (80-120% recovery).

### Fucosylated PSA

Lectin-based immunoassays for Fuc-PSA to detect AG PCa were developed and described previously 8. In this study, we used agarose bound AAL beads to enrich Fucosylated proteins from patient sera then tested PSA with the Hybritech PSA assay on the Access 2 Immunoassay Analyzer (Beckman Coulter, Inc.) 8, 18.

### PSA and *phi* analysis

Serum samples were analyzed for total PSA, free PSA (fPSA), and [-2]proPSA (p2PSA) 18, 21 on the Access 2 Immunoassay Analyzer (Beckman Coulter, Inc). Prostate health index (*phi*) was calculated with the equation, (p2PSA/fPSA) × PSA^1/2^.

### Statistical Analysis

Biomarker data were transformed prior to analysis (log-transformation followed by z-score). To correct for an observed batch-effect in Fuc-PSA measurement, z-scores of log-transformed Fuc-PSA data were computed separately for each of the two batches before being merged together. Scatterplots of the Fuc-PSA values before and after correction against total PSA, which was not affected by the batches, confirmed negligible residual differences (Figure S1). Furthermore, as shown in the same plots, with block-randomization of samples, the distribution of samples between the batches did not confound the sample clinical phenotype.

Diagnostic performance of individual biomarkers to differentiate AG from low risk PCa, and AG from low risk PCa and non-PCa were evaluated first by univariate analysis based on estimated AUCs from receiver-operating characteristic (ROC) curve analysis. To evaluate the statistical stability of results, bootstrap resampling (n = 1,000) 19, 22, 23 was used to estimate the mean and STD of AUCs of individual biomarkers.

Multivariate analyses were further carried out to evaluate the complementary values of biomarkers to established clinical test modalities with respect to the detection of AG PCa. With the limited number of available samples, we chose to evaluate only linear combinations using logistic regression of up to three novel markers with the clinical test *phi* and to identify panels of biomarkers with the greatest improvement in ROC/AUC over that of *phi* alone. This was done for both the detection of AG from low risk PCa, and from low risk PCa and non-PCa. In addition, we also specifically evaluated the value of Fuc-PSA in complementing *phi* as a two-marker panel. Bootstrap resampling was used to estimate 95% confidence intervals of ROC/AUCs.

Considering the potential clinical utility of a test to separate AG from low risk PCa (and/or non-PCa,), a very high sensitivity will likely be required to achieve a clinically acceptable negative predictive value for patient safety. For the identified multivariate panels, we therefore further assessed improvement in specificity at a fixed high level of sensitivity.

Differences between groups were assessed using the Mann-Whitney U test. Statistical significance was considered at *p*<0.05. Statistica 13 (StatSoft), GraphPad Prism 6 (GraphPad Software), MedCalc (MedCalc Software, Ostend, Belgium), and inhouse-developed Python scripts using library functions from matplotlib (2.2.3), NumPy (1.16.5), pandas (0.24.2), seaborn (0.9.0), scikit-learn (1.16.5) and SciPy (1.2.1) were used for statistical analyses. Other than specifically indicated, confidence intervals (CI) of AUCs and other performance measurement were based on bootstrap estimation.

## Results

### Patient characteristics

A total of 90 patients including 60 PCa cases and 30 non-PCa controls were included in this study. Among the PCa cases, 30 were biopsy GS ≤ 6 and the other 30 were GS ≥ 7. The non-PCa patients were biopsy negative controls. Among all the samples, one case was excluded due to a specimen quality issue. Of the remaining 89 samples, 7 had no PSA-related assay data and 2 had no Fuc-PSA data due to insufficient quantity for measurement. Consequently, other than the tabulated descriptive statistics and scatterplots of the individual biomarkers with the 89 samples, all statistical analyses were performed using 80 samples (25 AG and 25 low risk PCa, and 30 non-PCa) that had no missing data across all biomarkers.

Following an extended-pattern prostate biopsy schema 19, 98.8% of 80 patients underwent 12-core or greater biopsy with a median (range) number of 12 (8 to 20). Among 19 cases that went on to prostatectomy and had available pathologic GS, there were 3 cases with GS 6 upgraded to GS 7, and 3 with GS 8 and 1 with GS 9 downgraded to GS 7 on prostatectomy pathology. Detailed clinicopathologic characteristics of the study cohort, including diagnosis, age, race, family history of PCa, DRE (digital rectal examination), GS, clinical stage, PSA, %fPSA, and *phi* are shown in Table 1.

### Identification of biomarkers for multiplex immunoassay

In addition to evaluating two previously identified serum biomarkers (Fuc-PSA and Tie-2) 6-8, additional serum biomarkers with potential relevance to AG PCa were curated through a comprehensive literature search in PubMed. The inclusion of these biomarkers in our multiplex imunoassay panels took into consideration the reported clinically relevant performance characteristics and strength of evidence, biological feasibility supported by existing knowledge/databases such as results from large-scale genomic and proteomics analysis, ability to complement other biomarkers in the selection, their relative abundance in human serum samples, and the likelihood of available resources and constraints (antibodies, concentration in target specimens, etc.). Through *in silico* analysis, a total of 22 candidate biomarkers were selected to be assessed using a Bio-Plex 200 suspension array system (Bio-Rad) as described previously 24, 25 in 40 sera from patients diagnosed with AG or low risk PCa and benign prostate diseases, which were collected from JHH with institutional approval (data not shown). Ten candidate biomarkers (B7-H3, PLA2G7, GDF-15, IL-6 R alpha, SDC1, VCAM-1, IL-16, CA15-3, MMP-2 and HSP27) and one previously reported biomarker (Tie-2) were further evaluated using multiplex immunoassays in the 90 patient sera collected from Beth Israel Deaconess Hospital. The multiplex immunoassays had acceptable analytical performance with recoveries of 98% to 104%, intra-assay precision of 0.8% to 4.8%, inter-assay precision of 0.8% to 4.2%, wide dynamic concentration ranges (> 2 logs) defined by LLOQ and ULOQ, and low LOBs for target protein quantification (data not shown).

### Univariate evaluation of biomarker selection

**Figure 1:**
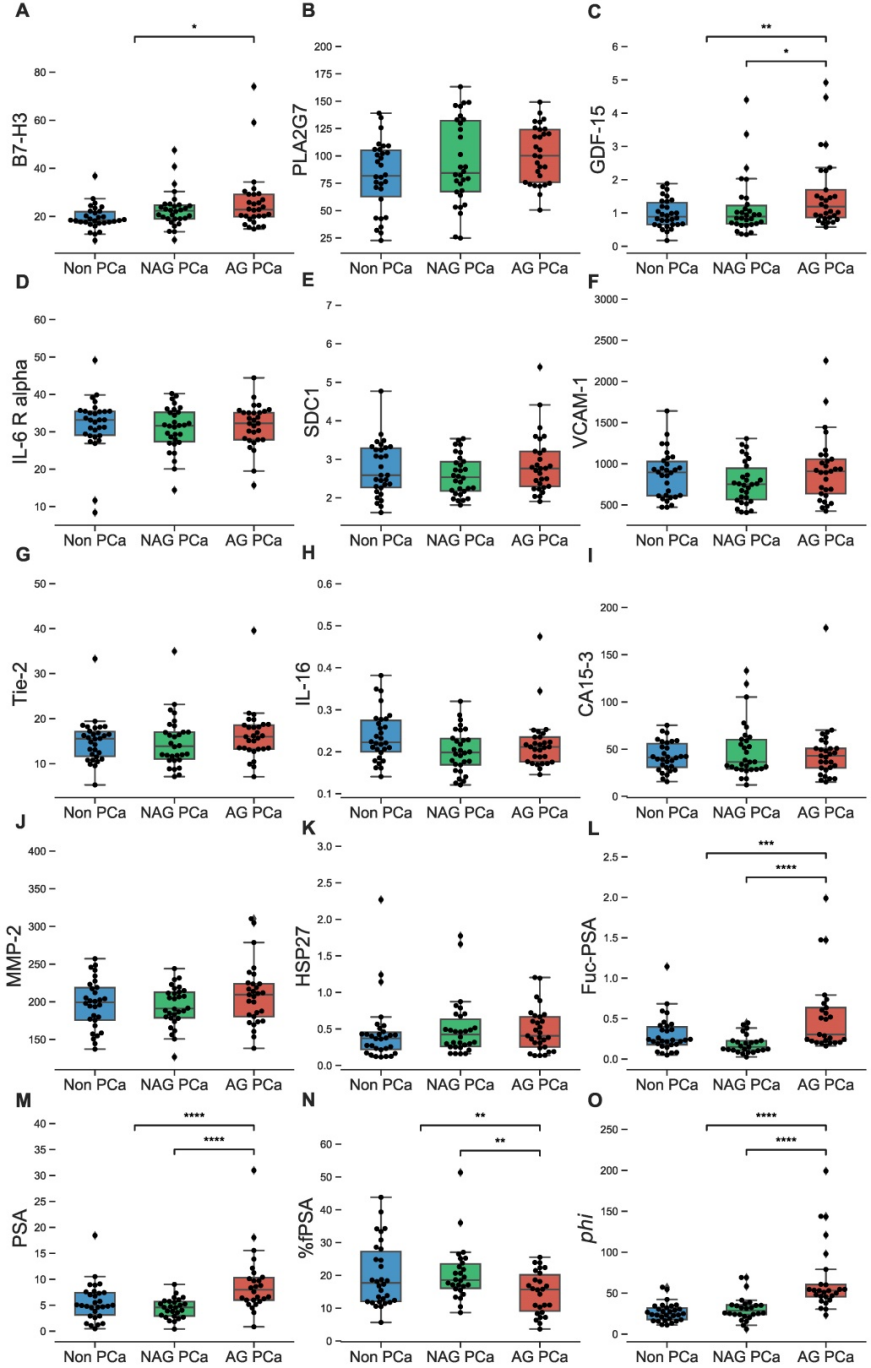
** Analysis of biomarkers in sera from NAG (low risk/non-aggressive) and AG PCa patients as well as biopsy negative controls.** A-O, B7-H3, PLA2G7, GDF-15, IL-6 R alpha, SDC1, VCAM-1, Tie-2, IL-16, CA15-3, MMP-2, HSP27, Fuc-PSA, PSA, %fPSA, and *phi* in NAG and AG PCa patients as well as biopsy negative controls (non-PCa) are demonstrated in overlaid scatterplots and boxplots. Only biomarkers demonstrating significant differences between AG and NAG PCa (or between AG and NAG + non-PCa) are shown with asterisks (Mann-Whitney *U* test). Bars in the boxes median value. *, *P* < 0.05; **, *P* < 0.01; ***, *P* < 0.001; ****, *P* < 0.0001.

Serum concentrations of individual biomarkers were compared among AG and low risk PCa patients as well as non-PCa controls (Figure 1A-O and Table S1). Biomarkers that individually showed a statistically significant difference in serum levels between AG and low risk PCa patients included GDF-15 (*p*<0.01), %fPSA (*p*<0.05, lower in AG), and Fuc-PSA, PSA, and *phi* (all at p<0.0001). When comparing AG PCa to the combined group of low risk PCa and non-PCa, biomarkers with significant differences included B7-H3 (*p*<0.05), %fPSA (*p*<0.05, lower in AG), GDF-15 (*p*<0.01), Fuc-PSA (*p*<0.001), and PSA and *phi* (both at *p*<0.0001).

**Figure 2:**
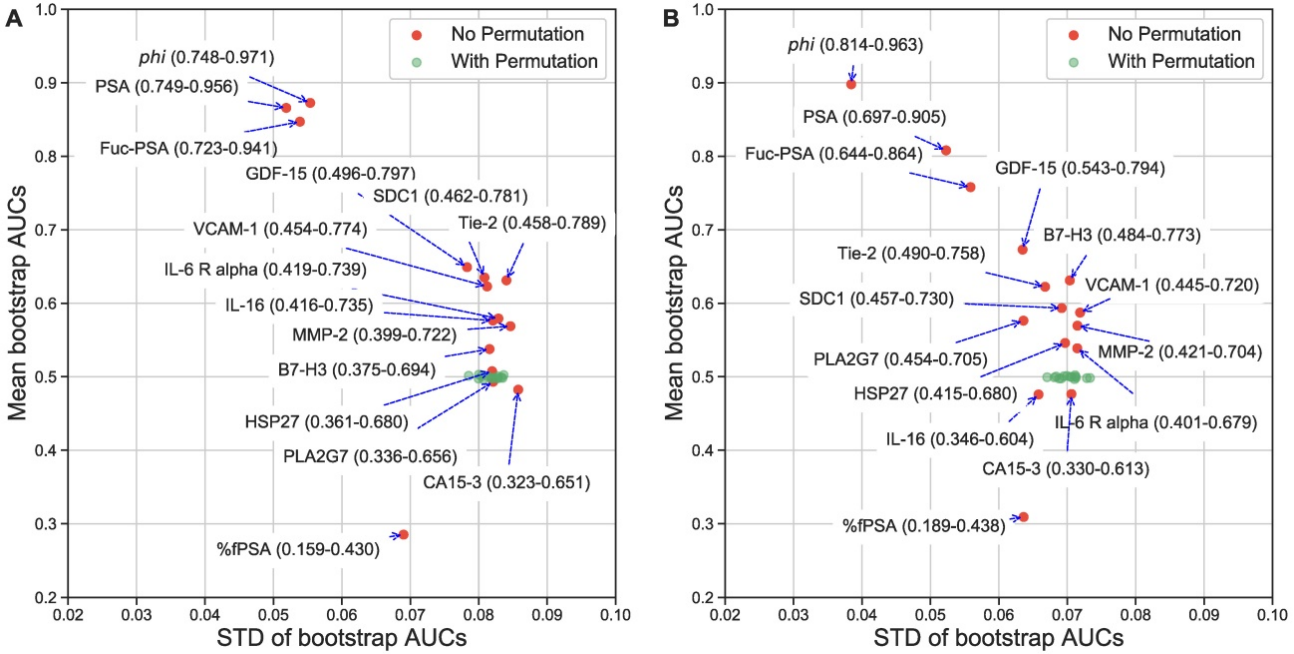
** Univariate evaluation of serum biomarkers.** Label permutation and bootstrap methods were used to evaluate statistical stability of the diagnostic performance of individual biomarkers in separating AG from NAG (low risk/non-aggressive) PCa (A) or NAG PCa and non-PCa (B). AUC means (95% CI) and STDs are presented.

To provide a more clinically relevant comparison, the AUCs from ROC analysis were also estimated. The best biomarkers to separate AG from low risk PCa were *phi* (AUC=0.872), PSA (0.866), Fuc-PSA (0.848), %fPSA (0.714]), GDF-15 (0.651), SDC1 (0.637), Tie-2 (0.635), and VCAM-1 (0.626), and to separate AG from low risk PCa and non-PCa were *phi* (AUC=0.898), PSA (0.807), Fuc-PSA (0.757), %fPSA (0.691), GDF-15 (0.673), B7-H3 (0.630), Tie-2 (0.620), and SDC1 (0.593). To further evaluate the statistical stability of biomarker performance within this sample set, Figures 2A and 2B show the bootstrap estimated mean and STDs for the AUCs of individual biomarkers. PSA related assays, including *phi*, had the best and most stable diagnostic performance in this specific cohort of patient samples.

### Multivariate evaluation of biomarker complementarity

In order to depict the strengths and relative relationships among the multiple biomarkers with respect to their ability to separate AG from either low risk PCa only or from low risk PCa and non-PCa, the biomarker data were used unsupervised through principal component analysis (PCA) to generate biplots 26 (Figure S2) in which the contributions (loadings) of individual biomarkers to the first and second principal components (PCs) were represented as vectors superimposed on the PCA plot of individual patient samples. As expected, when PSA, %fPSA, Fuc-PSA, and *phi* were included with the other candidate biomarkers in PCA analysis, the AG samples were reasonably well separated from either low risk PCa only (Figure S2A) or from low risk PCa and non-PCa (Figure S2B). Interestingly, for this particular sample set, there was no obvious separation between the low risk PCa and non-PCa samples. In the bioplots, the loading vectors of several non-PSA related candidate biomarkers, such as Tie-2, GDF-15, SDC1, B7-H7, VCAM-1 as a cluster, were at angles to those of the PSA-based biomarkers yet still pointed to the direction that would complement the PSA-based biomarkers in separating AG PCa and low risk PCa or non-PCa samples, indicating potential complementary value to the PSA-related tests. When a similar analysis was performed without the PSA-related assays, the clinical groups overlapped significantly (Figures S2C-D), with B7-H3, SDC1, GDF-15, Tie-2, and VCAM-1 retaining some level of contribution towards the separation of AG PCa samples. Tabulated pair-wise scatterplots (Figure S3A-B) of these biomarkers and the PSA-related biomarkers (nine in total) offer visualization of potential pair-wise complementary relations or the lack thereof among them.

**Figure 3:**
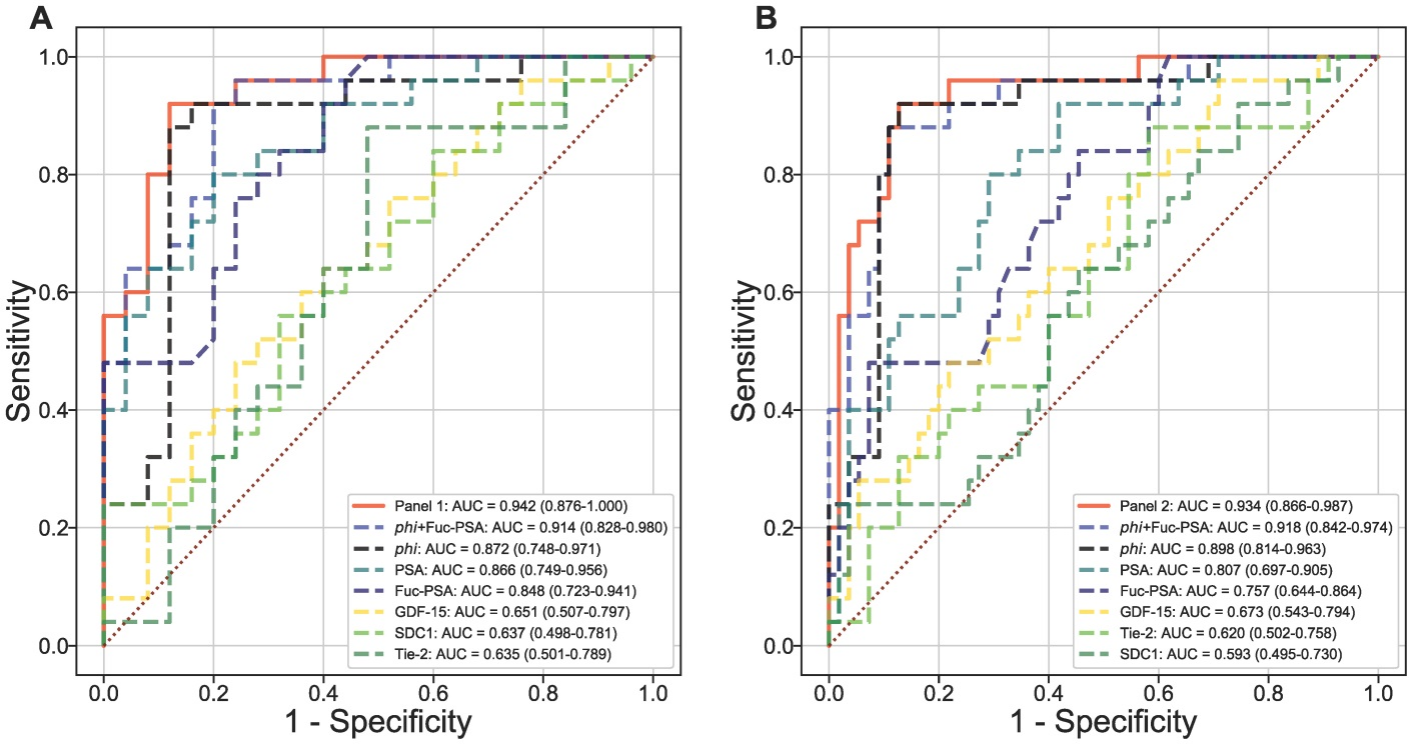
** Multivariate evaluation of serum biomarkers.** Diagnostic performance of combined serum biomarkers in separating AG from NAG (low risk/non-aggressive) PCa (A) or NAG PCa and non-PCa (B). ROC curves with AUCs are presented.

Using logistic regression, two panels were identified among all panels of up to 4 markers (including *phi* but excluding PSA and %fPSA) to offer the most improvement in ROC/AUC over that of *phi* alone in separating AG from low risk PCa (AUC*_phi_*_+Fuc-PSA+SDC1+GDF-15_ = 0.942 vs AUC*_phi_* = 0.872) or from low risk PCa and non-PCa (AUC*_phi_*_+Fuc-PSA+SDC1+Tie-2_ = 0.934 vs AUC*_phi_* = 0.898) (Figures 3A and 3B). In addition, the combination of *phi* and Fuc-PSA also improved the performance of *phi* in separating AG from low risk PCa (AUC*_phi_*_+Fuc-PSA_ = 0.914) or from low risk and non-PCa (AUC*_phi_*_+Fuc-PSA_ = 0.918). The improvement from these panels over *phi* were statistically significant comparing the means of bootstrap estimated AUCs (AUC*_phi_*_+Fuc-PSA+SDC1+GDF-15_ = 0.945 or AUC*_phi_*_+Fuc-PSA_ = 0.916 vs AUC*_phi_* = 0.873, both *p*<0.0001 for AG vs low risk PCa; and AUC*_phi_*_+Fuc-PSA+SDC1+Tie-2_ = 0.936 or AUC*_phi_*_+Fuc-PSA_ = 0.919 vs AUC*_phi_* = 0.898, both *p*<0.0001 for AG vs low risk PCa and non-PCa).

### The biomarker panels improved the specificity of AG PCa detection

For clinical applications, a very high sensitivity is required for the detection of AG PCa. In Table 2, to detect AG PCa from low risk PCa at a fixed sensitivity of 95.0%, the specificity of the four-marker panel of *phi*, Fuc-PSA, SDC1, and GDF-15, and the combination of *phi* and Fuc-PSA both had a specificity of 76.0% in comparison to that of 56.0% for *phi* (*p*=0.029, and 0.013, respectively) and 44.0% for PSA alone. Similarly, to detect AG PCa from low risk PCa and non-PCa at the same 95.0% sensitivity, the specificity for the four-marker panel of *phi*, Fuc-PSA, SDC1, and Tie-2, and the same *phi*, Fuc-PSA combinations had a specificity of 78.2% and 69.1%, respectively vs 65.5% for *phi* (*p*=0.010, and 0.207, respectively) and 36.4% for PSA alone.

## Discussion

Serum PSA has been used as a sensitive marker for the detection of PCa, but it is not confined to PCa, elevated serum PSA levels have also been observed in benign prostatic hyperplasia (BPH) and prostatitis 27, 28. Due to the potential for overdetection and overtreatment, PSA screening has caused controversy, posing a major challenge to the management of low-grade or low risk PCa 4. Overdetection associated with PSA screening highlights the urgent need to identify more efficient biomarkers with improved specificity. Such novel biomarkers or sophisticated PSA derivative tests may address the clinical dilemma of differentiating AG from clinically indolent low risk PCa, and help physicians to select patients for biopsy. *phi* is one of tools approved by the FDA to improve the detection of PCa. Compared with PSA, *phi,* which incorporates PSA, p2PSA and fPSA in the equation, enhances the specificity of PCa detection 19, 29 and has also shown to be associated with AG PCa. In this study, consistent with our previous studies 7, 8, Fuc-PSA confirmed its ability to separate AG from either low risk PCa only or low risk PCa and non-PCa. Combining Fuc-PSA with *phi* improved the detection of AG PCa from either low risk PCa or low risk PCa and non-PCa, both with statistical significance in a bootstrap comparison of AUCs. Furthermore, two four-marker panels of *phi*, Fuc-PSA, SDC1, and GDF-15 or *phi*, Fuc-PSA, SDC1, and Tie-2 were identified with an even greater improved performance over *phi* individually to separate AG from either low risk PCa or low risk PCa and non-PCa with statistical significance. Clinically more relevant, compared with *phi* alone, the four-marker panels significantly improved the specificity of AG PCa detection. Improvement in specificity at a fixed 95% sensitivity was also observed comparing the combination of *phi* and Fuc-PSA with *phi* alone.

In this study, we further validated two serum biomarkers previously discovered from our EDRN BRL studies 6-8, Fuc-PSA and Tie-2, as effective biomarkers for the detection AG PCa either as individual biomarkers or used in combination with other biomarkers. In addition, we also demonstrated the potential diagnostic value of two serum biomarkers, SDC1 and GDF15, in two four-marker panels that separate AG from either low risk PCa only or low risk PCa and non-PCa patients. Compared with our previous studies 6-8, we expanded the evaluation of the diagnostic value of candidate biomarkers in detecting AG not only from low risk PCa only but also from low risk PCa and non-PCa cases. Our results, if validated in patient cohorts representative of intended populations, could have the potential as an *in vitro* diagnostic multivariate index assay (IVDMIA) to provide valuable clinical information to help detect AG PCa.

There are limitations to our analysis, as the sample size of the current study was not sufficient for separate independent evaluation. However, within this sample set, bootstrap resampling provides evidence of statistical stability of the observed improvement. Additional studies will be needed for validation and to test the generalizability of the improvement in performance in independent samples.

The results observed in this study are consistent with other reports showing that specific glycoforms of PSA can potentially be used as biomarkers, not only to improve the diagnostic accuracy of PCa, but also to detect AG tumors 30-33. Changes in serum PSA sialylation have been reported in several studies 34-39, and specific increases in α2,3-sialic acid were observed in serum PSA in PCa patients compared with BPH and/or controls. In addition, increased core fucosylation of glycans has been detected in the serum of patients with PCa compared with healthy individuals or BPH 40, 41. Previously, we developed multiplex immunoassays, based on AAL lectin affinity capturing and protein-antibody immunoreactivity, to analyze serum fucosylated glycoproteins in PCa patients 7. Our data showed that Fuc-PSA was elevated and correlated with GS. Compared with total PSA, Fuc-PSA had better predictive ability to separate AG from low risk PCa. In addition, we previously developed two lectin-based immunoassays for the selection of glycoproteins containing fucosylated glycans using AAL and *Lens culinaris agglutinin* (LCA) followed by a clinical PSA immunoassay to analyze serum Fuc-PSA in PCa patients 8. Our data suggested that Fuc-PSA-AAL, and Fuc-PSA-LCA levels may be effective biomarkers to separate AG [particularaly for GS ≥ 7 (4+3)] from low risk PCa. AAL binds both core fucosylation and terminal fucosylation (α1-2/α1-3 fucosylation). In this study, we used agarose bound AAL beads to enrich Fuc-PSA from patient sera, therefore, the observed diagnostic value of serum Fuc-PSA in the detection of AG PCa could be attributed to both core fucosylation and terminal fucosylation of PSA, even though it has been reported that PSA fucosylation mainly occurs in the core glycan structure 42-44. Contrary to these results, Llop, et al reported that the core fucosylation level of serum PSA in high-risk PCa was significantly reduced compared to BPH and low-risk PCa, with an Enzyme-linked Lectin Assay (ELLA) including a double immunoprecipitation of serum PSA followed by *Phliota squarrosa lectin* (PhoSL) detection, which recognizes only core fucosylation 38. Contradictory reports on the glycosylation patterns of serum PSA may be attributed to a number of reasons. First, compared with antibodies, the binding affinity of lectins are much lower, and the concentration of PSA in patients's sera is very low, which makes the analysis of serum PSA glycosylation patterns very challenging, thus limiting the development of reliable assays with enough sensitivity for its detection in a large number of patient samples. Second, the analysis of serum PSA glycosylation patterns may be influenced by the glycosylated component present in complexed as opposed to free PSA forms. Lectins can bind not only to glycans on the target glycoproteins, but also to glycans on background glycoproteins (including antibodies), resulting in high background signals. Third, target and background glycoproteins might not be equally fucosylated, and multi-step sample preparation for glycan analysis could reduce quantitative accuracy and limit the analysis of a large number of patient samples in clinical studies to generate statistically significant data 31, 45.

Tie-2 is a transmembrane tyrosine kinase receptor for angiopoietins and plays a critical role in vascular development. It has been found to regulate the stemness and metastatic properties of PCa cells 17, and inhibiting angiopoietin-2 activity impedes angiogenesis and growth of LuCaP 23.1 PCa xenografts 16. Our previous study showed that the soluble Tie-2 levels in sera of PCa patients with GS of 8-10 were significantly increased, indicating that Tie-2 shedding might be related to the aggressiveness of PCa 6.

SDC1 is one of four structurally related cell surface heparan sulfate proteoglycans and plays a pivotal role in cell-cell and cell-extracellular matrix interactions 46. A significant increase in soluble SDC1 serum levels has been observed in advanced PCa cases, suggesting that SDC1 shedding might be related to PCa progression 47. In addition, elevated serum SDC1 was shown to be an independent factor in adverse overall and disease-specific survival in a multivariable pre-operative model, making evaluation of serum SDC1 levels a promising tool for pre-operative risk-stratification and/or therapy monitoring.

GDF-15, also known as macrophage inhibitory cytokine 1 (MIC-1), is a member of the transforming growth factor beta (TGF-ß) superfamily. It is synthesized as a 60-kDa dimer which is cleaved by furinlike proconvertases from its propeptide to release a 25-kDa mature protein 48. Only processed mature GDF-15 diffuses into the circulation, while the unprocessed, propeptide-containing form is frequently secreted from tumor cells and remains localized in tissues due to strong matrix binding mediated by its propeptide 48. Elevated serum GDF-15 levels have been found in many cancers, and shown to be a potentially valuable biomarker for cancer diagnosis and prognosis 25, 49, 50. The diagnostic complementarity between serum GDF-15 and PSA and/or %fPSA in the detection of PCa from BPH has also been reported 49, 51, 52. In this study, a significant increase in GDF-15 serum levels was observed in AG PCa cases compared with either low risk PCa or low risk PCa and non-PCa cases, which is consistent with the reports of elevated serum GDF15 in many cancers, including PCa 25, 49, 50. Stephan *et al,* has found that the levels of serum GDF-15 in benign disease was higher than that in PCa 52, but increased serum GDF-15 concentration was strongly associated with advanced disease and progression of PCa 50. Serum GDF-15 was found to be an independent marker of the presence of higher-grade (GS ≥ 7) tumors, which was not solely due to tumor burden. This observation is likely due to differences in processed GDF-15 or changed extracellular matrix properties 52.

Although the serum Tie-2 and SDC1 levels in patients with AG PCa were found to be elevated as compared to those with low risk PCa or low risk PCa and non-PCa, these differences were not statistically significant, likely due to the limited sample size. A logistic regression model was constructed to evaluate the ability of Fuc-PSA to further improve performance of *phi*. We then identified other contributing factors including SDC1, GDF-15, and/or Tie-2, and further evaluated the diagnostic performance of serum biomarker combinations in separating AG from low risk PCa only or low risk PCa and non-PCa cases. Compared with *phi* and PSA analysis, the multivariate panels showed clinically meaningful improvements. The selection of optimal panels through multivariate logistic regression allowed us to identify markers that are complementary in detecting AG PCa. However, to use these panels of serum protein biomarkers clinically as an IVDMIA assay, additional large-scale independent validation studies of these panels combined with other clinical and analytical parameters will be required. Recently, there has been an increased interest in the detection of tumor-specific molecular alternations by high-throughput screening - “omic” technologies. There are many promising biomarkers, including various tumor and serum proteins, microRNAs, as well as genetic markers that may be combined as diagnostic or prognostic indices 53.

In conclusion, through systematic proteomics analysis of multivariate combinations of serum biomarkers, we have identified panels of biomarkers that are potentially capable of detecting AG PCa, and demonstrated clinically meaningful improvement on the diagnostic performance of *phi*. It would be valuable to validate these panels in a large cohort of patient samples, because confounding factors such as age, body mass index (BMI), diabetes, and race may also affect the results. The multivariate combinations of serum biomarkers identified in this study warrant further clinical validation in a different and larger patient population, which could contribute to the clinical management of prostate cancer.

## Supplementary Material

Supplementary figures and tables.

## Figures and Tables

**Table 1:** Clinicopathologic characteristics of the study cohort

	Non-PCa	Low Risk PCa (GS 6)	AG PCa (GS ≥7)	All PCa	AG PCa breakdown by GS			
					GS 7 (3+4)	GS 7 (4+3)	GS 8	GS 9
Subjects, n (%)	30 (33.7)	30 (50.8)	29 (49.2)^§^	59 (66.3)^§^	4 (6.8)	6 (10.2)	9 (15.3)^§^	10 (16.9)
**Age (y)**								
mean ± SD	63.2 ± 8.6	61.3 ± 8.3	67.8 ± 9.9	64.5 ± 9.6	61.3 ± 7.6	66.7 ± 8.2	70.6 ± 11.4	68.5 ± 10.3
(range)	(43.0-80.0)	(46.0-77.0)	(51.0-93.0)	(46.0-93.0)	(51.0-69.0)	(56.0-77.0)	(51.0-93.0)	(55.0-87.0)
**Race, n (%)**								
White	29 (96.7)	25 (83.3)	29 (100.0)	54 (91.5)	4 (100.0)	6 (100.0)	9 (100.0)	10 (100.0)
Black	0 (0.0)	5 (16.7)	0 (0.0)	5 (8.5)	0 (0.0)	0 (0.0)	0 (0.0)	0 (0.0)
Asian	1 (3.3)	0 (0.0)	0 (0.0)	0 (0.0)	0 (0.0)	0 (0.0)	0 (0.0)	0 (0.0)
**FHx of PCa, n (%)**								
Yes	8 (26.7)	7 (23.3)	11 (37.9)	18 (30.5)	3 (75.0)	1 (16.7)	3 (33.3)	4 (40.0)
No	22 (73.3)	23 (76.7)	18 (62.1)	41 (69.55)	1 (25.0)	5 (83.3)	6 (66.7)	6 (60.0)
**DRE, n (%)**								
Abnormal	14 (46.7)	4 (13.3)	11 (37.9)	15 (25.4)	0 (0.0)	3 (50.0)	2 (22.2)	6 (60.0)
Enlarged	0 (0.0)	6 (20.0)	4 (13.8)	10 (17.0)	0 (0.0)	0 (0.0)	2 (22.2)	2 (20.0)
Normal	16 (53.3)	20 (66.7)	14 (48.3)	34 (57.6)	4 (100.0)	3 (50.0)	5 (55.6)	2 (20.0)
**Clinical Stage (T)**								
T1c/x	n/a	27/0 (90.0)	20/1 (72.4)	47/1 (81.4)	3/1 (100.0)	2/0 (33.3)	7/0 (77.8)	8/0 (80.0)
T2a/b/c/x	n/a	2/0/0/0 (6.7)	4/1/1/1 (24.1)	6/1/1/1 (15.3)	0/0/0/0 (0.0)	2/1/0/1 (66.7)	2/0/0/0 (22.2)	0/0/1/0 (10.0)
T3a	n/a	1 (3.3)	1 (3.5)	2 (3.3)	0 (0.0)	0 (0.0)	0 (0.0)	1 (10.0)
**PSA (ng/mL)**								
mean ± SD	5.58 ± 3.59	4.43 ± 1.92^*^	9.15 ± 5.84^†, #^	6.79 ± 4.92	7.43 ± 2.75	6.06 ± 2.11	12.48 ± 9.43^†^	9.00 ± 2.02^#^
median	4.97	4.58	7.98	5.79	6.67	5.99	11.35	9.17
(range)	(0.47-18.44)	(0.41-9.01)	(0.85-30.98)	(0.41-30.98)	(5.14-11.24)	(3.59-9.70)	(0.85-30.98)	(6.24-12.14)
**%fPSA**								
mean ± SD	20.19 ± 9.81	20.54 ± 8.56^*^	14.69 ± 6.50^†, #^	17.61 ± 8.08	15.91 ± 6.85	13.47 ± 5.89	16.00 ± 6.71^†^	13.69 ± 7.46^#^
median	17.7	18.56	15.68	17.1	16.25	13.26	16.4	12.57
(range)	(5.66-43.80)	(8.64-51.34)	(3.63-25.52)	(3.63-51.34)	(7.21-23.93)	(6.34-21.46)	(5.34-23.86)	(3.63-25.52)
***phi***								
mean ± SD	26.52 ± 11.23	31.30 ± 15.32^*^	66.19 ± 41.59^†, #^	48.74 ± 35.68	70.70 ± 49.18	52.35 ± 34.96	49.42 ± 9.61^†^	91.08 ± 54.49^#^
median	24.41	28.48	53.08	39.63	50.61	45.85	52.27	69.57
(range)	(11.04-57.27)	(6.09-69.10)	(23.18-199.18)	(6.09-199.18)	(37.72-143.84)	(23.18-120.95)	(30.41-60.74)	(41.58-199.18)

Note: PCa, prostate cancer. Non-PCa, biopsy negative; AG, aggressive; GS, Gleason score (biopsy); FHx, family history; DRE, digital rectal examination; *phi*, prostate health index. Median number of biopsy was 12 (range 8 to 20). PCa in 4 cases with GS 6 was upgraded on prostatectomy pathology. Original sample set n=90, one problematic sample with a specimen quality issue was omitted in estimation of descriptive analysis (§), additional missing data due to insufficient quantity for measurement were also indicated as *, #, and † for number of missing samples 4, 2, and 1, respectively.

**Table 2:** Biomarker panels improving the specificity of AG PCa detection

	AUC (95% CI)	SN (%)	SP (%) (p-value*)	True-Neg	True-Pos	False-Neg	False-Pos
**AG vs Low Risk PCa**							
Panel-1	0.942 (0.876-1.000)	95.0	76.0 (0.029)	19	24	1	6
*phi* + Fuc-PSA	0.914 (0.828-0.980)	95.0	76.0 (0.013)	19	24	1	6
*phi*	0.872 (0.748-0.971)	95.0	56.0	14	24	1	11
PSA	0.866 (0.749-0.956)	95.0	44.0	11	24	1	14
**AG vs Low Risk PCa & Non-PCa**							
Panel-2	0.934 (0.866-0.987)	95.0	78.2 (0.010)	43	24	1	12
*phi* + Fuc-PSA	0.918 (0.842-0.974)	95.0	69.1 (0.207)	38	24	1	17
*phi*	0.898 (0.814-0.963)	95.0	65.5	36	24	1	19
PSA	0.807 (0.697-0.905)	95.0	36.4	20	24	1	35

**Note**: PCa, prostate cancer; AG, aggressive PCa; Non-PCa, biopsy negative; Panel-1, *phi* + Fuc-PSA + SDC1 + GDF-15; Panel-2, *phi* + Fuc-PSA + SDC1 + Tie-2; AUC, area under curve; CI, confidence interval; SN, sensitivity; SP, specificity; Neg, negative; Pos, positive. *, one-sided paired test comparing specificity against *phi*.

## Abbreviations

AG: aggressive
CA15-3: cancer antigen 15-3
B7-H3: CD276 molecule
DRE: digital rectal examination
EDRN: Early Detection Research Network
fPSA: free PSA
Fuc-PSA: fucosylated PSA
GS: Gleason score
GDF-15: growth differentiation factor 15
HSP27: heat shock 27 kDa protein
IL-16: interleukin 16
IL-6 R alpha: interleukin-6 receptor subunit alpha
MMP-2: matrix metallopeptidase 2
NIH/NCI: National Institutes of Health/National Cancer Institute
NAG: non-aggressive
PLA2G7: phospholipase A2 group VII
PCa: prostate cancer
phi: Prostate Health Index
PSA: prostate-specific antigen
p2PSA: [-2]proPSA
SDC1: Syndecan-1
Tie-2: TEK receptor tyrosine kinase
VCAM-1: vascular cell adhesion molecule 1
