# Supplementary material for: A panel of selected serum protein biomarkers for the detection of aggressive prostate cancer
Source: Theranostics. 2021 Apr 15;11(13):6214–24. doi: 10.7150/thno.55676 (PMC8120218; doi:10.7150/thno.55676)
Supplement: Supplementary file 1 — Supplementary figures and tables. [file thnov11p6214s1.pdf]

Supplement Figure Legends

Figure S1. Batch effect identification and correction. A. Distributions of Fuc-PSA in the two batches (Runs 1, 2 and Runs 3, 4, 5). **B**. Normalized distributions of Fuc-PSA in the two batches.

Figure S2. PCA biplot of individual serum biomarkers. PCA biplot combined with PCA score plot and loading plot was used to analyze individual serum biomarkers for classifying patients with AG and NAG (low risk/non-aggressive) PCa only (**A & C**) or classifying AG and NAG PCa patients as well as biopsy negative controls (**B & D**), demonstrating clusters of samples based on their similarity (PCA score plot) and how strongly each characteristic influences a principal component (i.e. PC-1 and PC-2).

Figure S3. The scatterplot matrix of selected serum biomarkers. The complementarities of 9 selected serum biomarkers in separate AG either from NAG (low risk/non-aggressive) PCa only (**A**) or NAG PCa and non-PCa (**B**) are demonstrated.

Table S1. Biomarker descriptive statistics.

Biomarker	Subgroup	Number	Min	Max	Median	Mean	STD	IQR
**B7-H3**	Non-PCa	30	9.99	36.81	18.36	19.50	5.05	4.58
	Low Risk PCa	30	10.24	47.52	22.31	22.87	7.56	5.70
	AG PCa	29[a]	14.77	74.01	22.85	25.84	12.60	9.25
**PLA2G7**	Non-PCa	30	22.68	139.06	81.81	81.72	31.90	42.36
	Low Risk PCa	30	24.87	163.22	84.32	94.21	38.96	64.88
	AG PCa	29 [a]	50.49	149.22	100.23	101.59	25.97	48.26
**GDF-15**	Non-PCa	30	0.17	1.88	0.89	0.98	0.43	0.66
	Low Risk PCa	30	0.35	4.40	0.89	1.17	0.89	0.55
	AG PCa	29 [a]	0.58	4.92	1.19	1.57	1.10	0.83
**IL-6 R alpha**	Non-PCa	30	8.39	49.13	33.15	31.91	7.49	6.39
	Low Risk PCa	30	14.40	40.22	31.61	30.78	5.97	7.83
	AG PCa	29 [a]	15.67	44.42	32.25	31.62	5.84	7.25
**SDC1**	Non-PCa	30	1.62	4.77	2.59	2.75	0.70	1.02
	Low Risk PCa	30	1.81	3.54	2.54	2.58	0.52	0.76
	AG PCa	29 [a]	1.90	5.40	2.76	2.87	0.77	0.91
**VCAM-1**	Non-PCa	30	471.35	1641.00	894.76	866.64	286.83	414.75
	Low Risk PCa	30	406.97	1306.00	752.16	773.53	259.86	379.87
	AG PCa	29 [a]	424.37	2251.80	908.17	927.48	403.58	419.38
**Tie-2**	Non-PCa	30	5.27	33.29	15.54	14.98	4.85	5.48
	Low Risk PCa	30	7.12	34.95	13.89	14.83	5.76	5.98
	AG PCa	29 [a]	7.10	39.54	16.00	16.29	5.72	5.30
**IL-16**	Non-PCa	30	0.14	0.38	0.22	0.24	0.06	0.07
	Low Risk PCa	30	0.12	0.32	0.20	0.20	0.05	0.06
	AG PCa	29 [a]	0.15	0.47	0.21	0.22	0.06	0.06
**CA15-3**	Non-PCa	30	15.55	75.39	41.32	42.52	15.54	24.96
	Low Risk PCa	30	12.09	132.83	36.44	48.45	29.25	30.95
	AG PCa	29 [a]	15.24	178.28	42.92	45.88	30.12	20.74
**MMP-2**	Non-PCa	30	137.13	257.18	199.19	196.91	32.48	42.89
	Low Risk PCa	30	126.74	244.01	190.98	194.09	26.48	33.86
	AG PCa	29 [a]	138.27	310.22	209.41	208.79	40.79	43.37
**HSP27**	Non-PCa	30	0.12	2.27	0.37	0.45	0.43	0.23
	Low Risk PCa	30	0.16	1.77	0.42	0.51	0.39	0.37
	AG PCa	29 [a]	0.14	1.20	0.41	0.49	0.30	0.41
**Fuc-PSA***	Non-PCa	30	0.05	1.14	0.23	0.30	0.23	0.22
	Low Risk PCa	25 [b]	0.03	0.45	0.15	0.19	0.12	0.12
	AG PCa	25 [a, b]	0.16	1.99	0.30	0.54	0.47	0.41
**PSA**	Non-PCa	30	0.47	18.44	4.97	5.58	3.59	4.26
	Low Risk PCa	26 [b]	0.41	9.01	4.58	4.43	1.92	2.76
	AG PCa	26 [a, b]	0.85	30.98	7.98	9.15	5.84	4.34
**%fPSA**	Non-PCa	30	5.66	43.80	17.70	20.19	9.81	15.16
	Low Risk PCa	26 [b]	8.64	51.34	18.56	20.54	8.56	7.41
	AG PCa	26 [a, b]	3.63	25.52	15.68	14.69	6.50	11.04
phi	Non-PCa	30	11.04	57.27	24.41	26.52	11.23	13.83
	Low Risk PCa	26 [b]	6.09	69.10	28.48	31.30	15.32	11.63
	AG PCa	26 [a, b]	23.18	199.18	53.08	66.19	41.59	14.90

NOTE: all biomarkers are in ng/mL, except for CA15-3 in U/mL, %fPSA as a percentage, and *phi* with no units. IQR: interquartile range. Non-PCa: biopsy negative. Low Risk PCa: GS ≤ 6. AG PCa, GS ≥ 7. [*], considering the statistical difference between the two batches, we first calculated the Spearman correlation between the Fuc-PSA and PSA values in the two batches, and used the batch with the higher correlation as the baseline, and then used the other batch normalized to the same baseline mean and standard deviation. [a, b], missing data due to either 1 problematic sample with a specimen quality issue or 9 samples without sufficient quantity for the measurements.

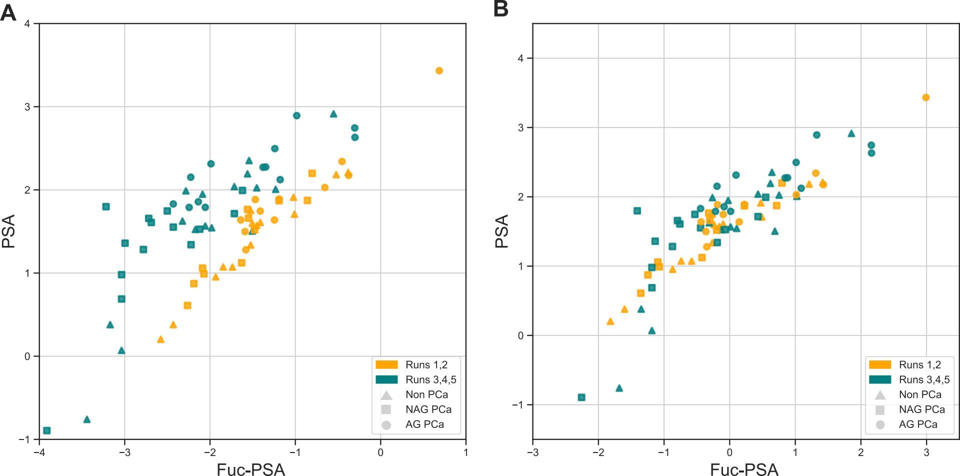
A
B
PSA
Fuc-PSA
Runs 1,2
Runs 3,4,5
Non PCa
NAG PCa
AG PCa
−4
−3
−2
−1
0
1
2
3
4

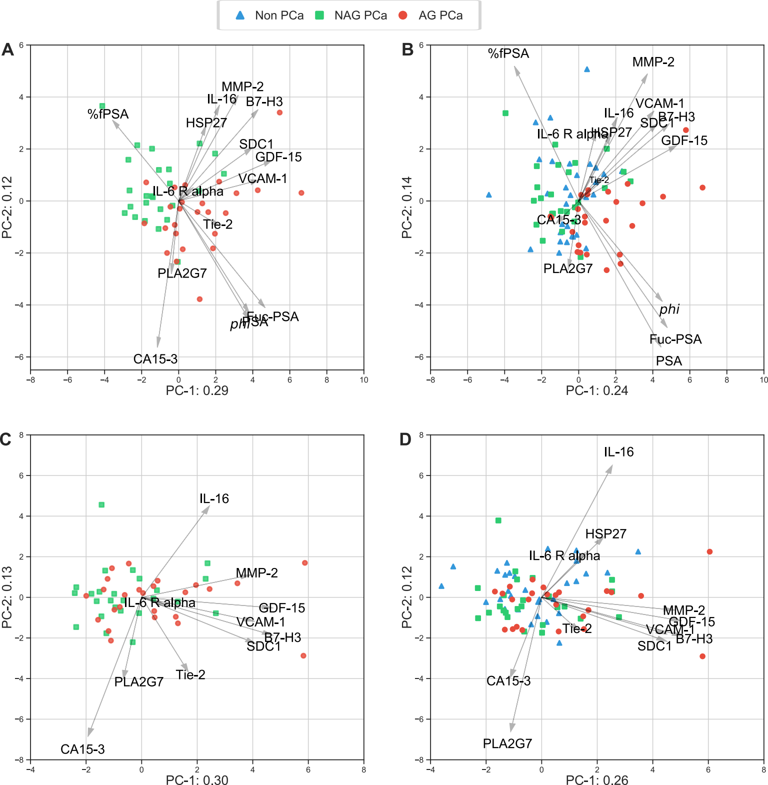
Non PCa
NAG PCa
AG PCa
A
PC-1: 0.29
PC-2: 0.12
%fPSA
MMP-2
IL-16
B7-H3
HSP27
SDC1
GDF-15
VCAM-1
IL-6 R alpha
Tie-2
PLA2G7
Fuc-PSA
phi
PSA
CA15-3
B
PC-1: 0.24
PC-2: 0.14
%fPSA
MMP-2
VCAM-1
IL-16
B7-H3
SDC1
IL-6 R alpha
HSP27
GDF-15
Tie-2
CA15-3
PLA2G7
phi
Fuc-PSA
PSA
C
PC-1: 0.30
PC-2: 0.13
IL-16
MMP-2
IL-6 R alpha
GDF-15
VCAM-1
B7-H3
SDC1
PLA2G7
Tie-2
CA15-3
D
PC-1: 0.26
PC-2: 0.12
IL-16
HSP27
IL-6 R alpha
MMP-2
GDF-15
VCAM-1
B7-H3
SDC1
Tie-2
CA15-3
PLA2G7

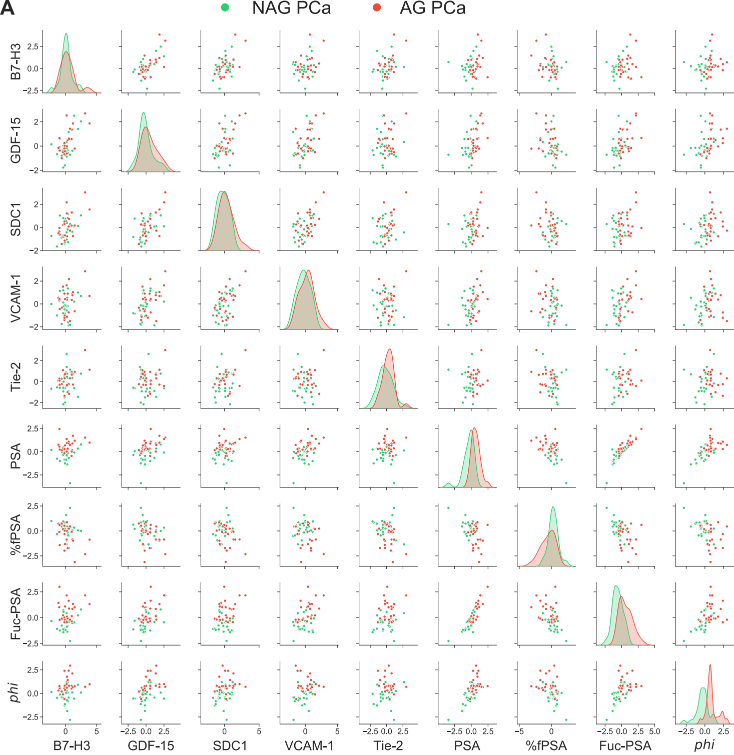
A
NAG PCa
AG PCa
B7-H3
GDF-15
SDC1
VCAM-1
Tie-2
PSA
%fPSA
Fuc-PSA
phi

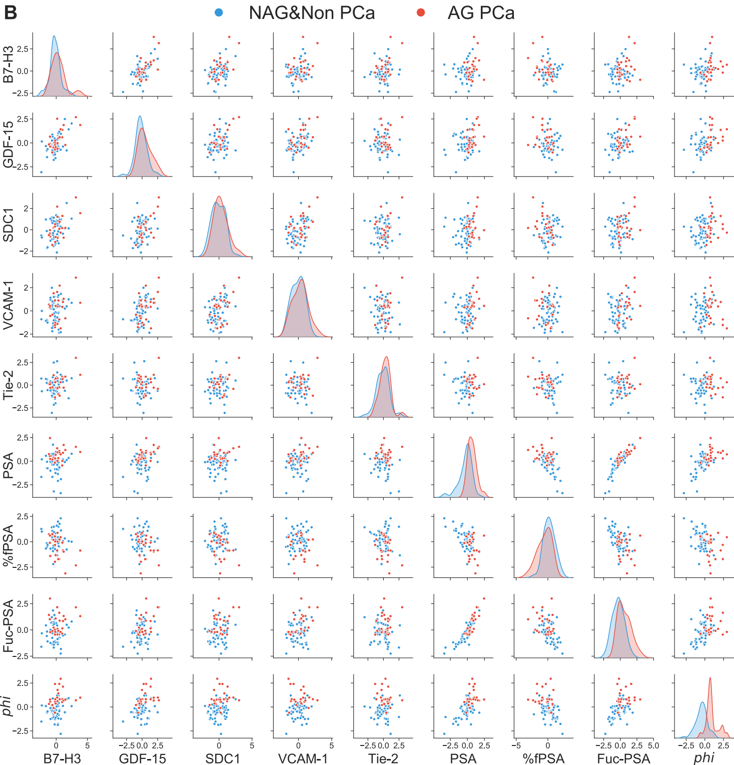
B
NAG&Non PCa
AG PCa
B7-H3
GDF-15
SDC1
VCAM-1
Tie-2
PSA
%fPSA
Fuc-PSA
phi
